# Evaluation of Larvicidal Activity of Essential Oil from Leaves of *Coccinia grandis* against Three Mosquito Species

**Published:** 2017-05-27

**Authors:** Shahid Iqbal Mohammed, Kishor Sukhlal Vishwakarma, Vijay Laxminarayan Maheshwari

**Affiliations:** School of Life Sciences, North Maharashtra University, Jalgaon, India

**Keywords:** *Coccinia grandis*, Essential oil, Mosquito, Larvicidal, GC-MS

## Abstract

**Background:**

To study the chemical constituents and larvicidal activity of essential oil extracted from the leaves of *Coccinia grandis* against three mosquito species.

**Methods:**

Essential oil was extracted by hydro distillation using clevenger apparatus and was analyzed for chemical constituents by gas chromatography-mass spectrophotometry (GC-MS). Larvicidal activity was recorded after 12 and 24h of post-exposure against three mosquito species, *Anopheles stephensi*, *Aedes aegypti* and *Culex quinquefasciatus*. Dead larvae were identified when they failed to move after probing with a needle in the siphon or cervical region. The LC_50_ and LC_90_ values for three mosquito larvae were calculated by Probit analysis.

**Results:**

The GC-MS analysis revealed that essential oil contains 23 different constituents. Out of these 23 constituents, major constituents identified were n-tetracosane (39.18%), n-eicosane (30.04%), tetratriacotane (2.97%), 7-octadecanal (2.81%), and tricosane (2.31%). Essential oil from leaves of *Coccinia grandis* exhibited significant larvicidal activity against *An. stephensi* with LC_50_ and LC_90_ values 39.41ppm and 123.24ppm, respectively. This was followed by *Ae. aegypti* and *Cx. quinquefasciatus* with LC_50_ and LC_90_ values of 48.20ppm, 131.84ppm and 52.80ppm, 135.48ppm, respectively after 24h of exposure.

**Conclusion:**

The results could be useful in developing a cost effective, ecofriendly, region specific and practical strategy for the control of mosquito vectors.

## Introduction

Mosquitoes are responsible for a number of human health problems causing illness and death throughout the world in both children and adults. They are vector for many diseases such as malaria, filariasis, dengue, Japanese encephalitis, chikungunya and west Nile virus infection in tropical and subtropical countries (Anupam et al. 2012). Malaria is a deadly disease and globally about 3.3 billion peoples are at the risk of it. About 198 million cases of malaria and 0.58 million deaths occurred globally in 2013 (WHO 2014). Of the six malarial vector species, *Anopheles stephensi* is the main mosquito vector responsible for malaria in urban areas of India (Senthilkumar et al. 2009). Dengue fever is caused by mosquito vector species, *Ades aegypti* in its epidemic areas affecting millions of people and thousands of deaths per year all over the world (Service 1996). Similarly, *Culex quinquefasciatus* is a vector for lymphatic filariasis, commonly known as elephantiasis, in India. Lymphatic filariasis is caused by the worms *Wuchereria bancrofti*, *Brugia malayi* and *Br. timori* of which, first is found to be more endemic in Indian subcontinent. According to WHO (2009) more than 1.3 billion people spread over 72 different countries worldwide are threatened by it. Collectively, these mosquito mediated diseases are responsible for long term suffering, morbidity and high socio economic burden on society (Ramaiah et al. 2000, Intirach et al. 2012).

Efficient control of these diseases would require a two-pronged strategy, (i) prompt treatment with effective medicines and (ii) vector control based prevention strategies. Injudicious use of medicines, particularly antimalarial drugs, has resulted in development of resistance by the malarial parasite and enhanced casualties in endemic areas (Intirach et al. 2012). Mosquito management therefore, offers a better and practical alternative for controlling diseases mediated by them. Mosquito adulticides, causing temporary reduction in population are good but do not offer a lasting solution. Mosquito larvae being delicate, less mobile and more concentrated in their natural habitat offer a much simpler and efficient point of intervention and control (Rutledge et al. 2003, Dharmagadda et al. 2005). However, there are reports of development of resistance and behavior changes in adult mosquitoes and larvae towards these chemicals (Mittal et al. 2004, WHO 2006). Moreover, they adversely affect the environment, causing soil, air, water pollution and harm the beneficial non target organisms (Dharmagadda et al. 2005). Plant extract including essential oils or insecticides from botanical origin are attractive alternatives because they contain high amount of various bioactive compounds, many of which are selective and have little or no harmful effects on non-target organisms and environment (Rutledge et al. 2003, Dharmagadda et al. 2005). Essential oil is natural volatile substances found in many plants. Essential oils isolated from plant are generally a mixture of many constituents, primarily biologically active monoterpenes (Govindrajan 2010). Traditionally, they have been used for flavor enhancement in food, odorants in fragrances, pharmaceuticals and confectionary industries (Zhu et al. 2001). Of late, they have received considerable attention as potentially active, human and environment friendly bio insecticides (Cheng et al. 2003). There are several reports on larvicidal activity of essential oil from neem, basil, citronella, lemon, eucalyptus, pine etc (Cheng et al. 2003, Amer and Mehlhorn 2006, Dua et al. 2009). The resistance against plant derived insecticides has not been reported so far (Kannathasan et al. 2011).

*Coccinia grandis* (family Cucurbitaceae) is a unique tropical plant, commonly known as ‘little gourd’, growing abundantly and widely all over the India. It is a fast growing perennial climbing shrub with white flowers. It grows several meters long and forms dense mat that readily cover shrubs and small trees. It is well known for its hypoglycemic activity (Ajay 2009, Munasinghe et al. 2011).

The present study was focused on the chemical constituents and larvicidal activity of *Coccinia grandis* leaf essential oil against vectors of malaria (*An. stephensi*), dengue (*Ae. aegypti*) and filariasis (*Culex quinquefasciatus*). To the best of our knowledge it is the first report on the larvicidal activity of *Coccinia grandis* leaf essential oil against the three mosquito species.

## Materials and Methods

### Collection of Plant material and extraction of essential oil

Plant material of *Coccinia grandis* was collected from Eklagna village Jalgaon. [20° 58′ 54.3″ N, 075° 27′ 09.5′ E (elevation: 199m)] Maharashtra, identified at Botanical Survey of India, Pune and a specimen voucher number MSMI-1 was deposited in the School of Life Sciences, North Maharashtra University Jalgaon. The collected fresh leaves were cut in to small pieces and extraction was done using Clevenger apparatus for 6h (Singh et al. 2008). The extracted essential oil was subjected to dryness over anhydrous sodium sulfate (Na_2_SO_4_) to remove traces of moisture. The physical characteristics of extracted essential oil were recorded, percentage average yield was calculated and it was stored at 4 °C in amber-colored bottle in refrigerator until further analysis.

### GC and GC-MS analysis

Gas chromatography mass spectroscopy (GC-MS) analysis of essential oil was performed using JEOL GCMS-Mate-II model gas chromatograph-mass spectrometer equipped with an AOC-20i auto injector and HP-5 capillary column (30m × 0.25mm ID × 0.25μm coating thickness) column. The injector temperature was set at 280 °C, and the oven temperature was initially set at 40 °C then programmed to increase up to 300 °C at the rate of 10 °C/min and finally held at 200 °C for 5 min. Helium gas was maintained at a flow rate 1.0ml/min as a carrier gas. One microliter of the sample diluted with acetone in 1:10 ratio was injected in the split mode. The percentage of constituents in essential oil of leaves was calculated by the GC peak areas. Data handling was made through JEOL software and the compounds were identified based on the comparison of their retention time (RT) and mass spectra of WILEY, NIST library data of the GC-MS.

### Mosquito larvicidal assay

Mosquito larvicidal activity was performed against mosquito larvae of species *An. stephensi*, *Ae. aegypti* and *Cu. quinquefasciatus.* Larvae of *An.s stephensi* [21^0^00′14.3N, 075^0^29′39.8E (elevation: 207m)], *Ae. aegypti* [2101′01.2N, 075^0^29′52.3E (elevation: 192m)] and *Cu. quinquefasciatus* [21^0^00′53.5N, 075^0^ 29′39.8E (elevation: 185m] were collected from local breeding areas of Jalgaon, India and identified using the microscopic examination as per Theodore et al. (2005). The collected mosquito larvae were brought to laboratory and maintained at 25–30 °C with 80–90% relative humidity and 12 h/d/night cycle in plastic trays containing dechlorinated water. Mosquito larvae were fed with 10% sterile sucrose solution and pet biscuits. The mosquito larvicidal activity was performed according to standard procedure recommended by WHO (1981). The extracted dried and pre weighed essential oil was dissolved in 1ml of acetone and from this different concentrations were made such as 3.125, 6.25, 12.50, 25, 50 and 100ppm in distilled water. Twenty five early fourth instar stage larvae of each of the three species of mosquito were used for larvicidal assay in 200ml beakers and three replicates were maintained for each concentration used. During the experiment, no food was given to the larvae.

### Statistical analysis

The larval mortality rate was calculated after 12 and 24 h of exposure time. The lethal concentrations, LC_50_ and LC_90_ and their 95% confidence limit of the lower and upper levels were calculated by probit analysis using statistical software Stats Direct 2.8.0.

## Results

**Fig. 1.**
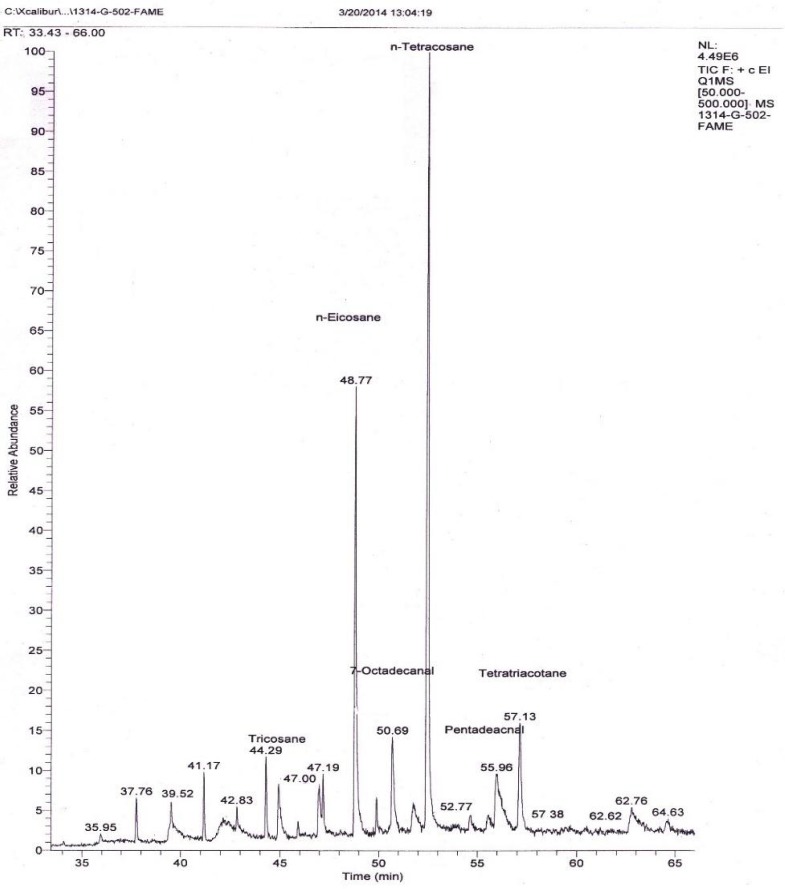
Gas chromatography–mass spectrometry profile of essential oil obtained from leaves of *Coccinia grandis*

The essential oil yield from fresh and finely cut leaves of *Coccinia grandis* was 0.14gm% (*w/w*). The yield was calculated after drying (removing the moisture) over anhydrous sodium sulfate (Na_2_SO_4_). The essential oil after dryness gave a slightly sticky clump with light yellow color and a characteristic odor. The GC-MS profile of the essential oil from leaves of *Co. grandis* is shown in Fig. 1. The various constituents of essential oil, their retention time and percent composition in order of elution from the column are given in the Table 1. The GC-MS profile shows a total of 23 constituents accounting for 99.60% of total oil. The two major constituents of essential oil from leaves of *Co. grandis* were n-tetracosane (39.18) and n-eicosane (30.04%). Six constituents (peak number 4, 6, 10, 13, 18 and 19) were present between 2–3 percent were as the percentage composition of remaining ranged between 0.1–2 percent (Table 1).

**Table 1.** Chemical composition of essential oil of the leaves of *Coccina grandis.*

**Peak no.**	**Retention time(min)**	**Chemical compounds**	**Percentage**
**01.**	35.95	E,E,Z-1,3, 12-Nanodecatriene-5,14-diol	0.81
**02.**	37.76	Heneicosane	1.34
**03.**	39.52	Phytol	1.35
**04.**	41.17	1-heptatriacotanol	2.06
**05.**	42.83	17-pentatriacontene	1.19
**06.**	44.29	Tricosane	2.31
**07.**	45.16	1- Dodecanol, 2-Coctyl-	1.37
**08.**	45.96	2,5-Furandione, 3-dodecyl	0.96
**09.**	47.00	Tetrapentacosane	1.98
**10.**	47.19	2-Dodecen-1-yl(-)sucinic anhydride	2.08
**11.**	48.77	n-Eicosane	30.04
**12.**	49.21	Octasane	1.37
**13.**	50.69	7-octadecanal	2.81
**14.**	51.06	Hexatriacontane	1.23
**15.**	52.77	n-tetracosane	39.18
**16.**	54.74	1,3 O-triacotanediol	1.09
**17.**	55.41	Z-14-octadecen-1-ol acetate	0.98
**18.**	55.96	Pentadeachal	2.09
**19.**	57.13	Tetratriacotane	2.97
**20.**	57.38	Triacotane	0.11
**21.**	62.62	Meissyl alcohol	0.13
**22.**	62.76	Palmitic acid	1.23
**23.**	64.63	Myristic acid	0.92

	**Total**		**99.60%**

The essential oil extracted from leaves of *Co. grandis* shows promising larvicidal activity against three mosquito species *An. stephensi*, *Ae. aegypti* and *Cu. quinquefasciatus*, (Table 2). The LC_50_ and LC_90_ values against early fourth instar larvae of *An. stephensi*, after 12 and 24 h of exposure were calculated to be 72.60 and 169.90 and 39.41 and 123.24ppm, respectively. Similarly, LC_50_ and LC_90_ values against early fourth instar larvae of *Ae. aegypti* after 12 and 24h of exposure were calculated to be 83.25 and 191.60 and 48.20 and 131.84ppm, respectively. The values were marginally higher with early fourth instar larvae of *Cx. quinquefasciatus* than the other two species under identical conditions (Table 2).

**Table 2.** Larvicidal activity of *Coccinia grandis* leaf essential oil after 12 and 24 h of exposure period on larvae of *Anopheles stephensi*, *Aedes aegypti* and *Culex quinquefasciatus*

**Mosquito species**	**Time**	**Concentration (ppm)**	**% of Mortality±SE**	**LC_50_ (LCL–UCL)^a^**	**LC_90_ (LCL–UCL)^a^**	**X^2^ (df=4)^b^**
***An. stephensi***	After 12h	3.125	8.0±6.89	72.60 (43.12–106.50)	169.90 (90.05–265.96)	12.60

	After 24h	6.25	18±5.77	39.41 (12.07–67.619)	123.24 (43.276–212.45)	28.581
		12.5	25±1.25			
		25	35±1.52			
		50	42±1.55			
		100	60±0.57			
		3.125	17±0.21			
		6.25	21±0.82			
		12.5	41±1.00			
		25	55±1.52			
		50	66±1.20			
		100	75±1.85			

***Ae. aegypti***	After 12h	3.125	7.0±0.33	82.35 (25.73–145.96)	191.60 (35.89–370.94)	27.077

	After 24h	6.25	11±0.87	48.20 (19.25–78.66)	131.84 (49.23–223.96)	27.862
		12.5	26±0.21			
		25	38±1.52			
		50	43±0.57			
		100	51±0.29			
		3.125	14±1.20			
		6.25	17±0.88			
		12.5	33±0.86			
		25	51±0.68			
		50	61±1.15			
		100	71±0.26			

***Cx. quinquefasciatus***	After 12h	3.125	5.0±0.28	100.40 (36.18–175.94)	217.39 (54.32–413.03)	19.41

	After 24h	6.25	10±0.39	52.805 (23.92–83.50)	135.48 (55.54–224.83)	25.756
		12.5	20±0.57			
		25	31±1.20			
		50	36±0.21			
		100	41±0.28			
		3.125	12±0.13			
		6.25	16±0.57			
		12.5	29±0.39			
		25	47±1.20			
		50	59±0.92			
		100	69±0.96			

^a^Degree of freedom

LCL lower confidence level,

UCL upper confidence level

^a^95% confidence level

## Discussion

Several authors have reported different compositions of essential oils obtained from different plant species (Govindrajan 2010, Senthilkumar and Venkatesalu, 2010, Zhu et al. 2011, 2012, Rajkumar et al. 2011, Intirach et al. 2012, Liu et al. 2012 and Senthilkumar et al. 2013). The plants show significant variations, both in terms of number and percentage composition, of different constituents in essential oil and it appears to be the characteristics of a particular plant.

The excellent larvicidal activity of the essential oil of *Co. grandis* against three species of mosquitoes could be either due to the major components, i.e. n-tetracosane and eicosane or synergistic action of the major and minor components present in it and is difficult to pin point at this stage. Tetracosane is an alkane hydrocarbon and use of hydrocarbons as pesticidal agents is reported (Siddique et al. 2004). Similarly, there are evidences indicating larvicidal and antimicrobial activities of the other major component, eicosane (Akpuaka et al. 2013, Manas et al. 2014). The results show significantly improved bioefficacy against one of the mosquito species, *An. stephensi* compared to earlier report which showed LC_50_ and LC_90_ values of 93.3 and 192.6ppm, respectively (as against the 39.41 and 123.24ppm in present study) (Rajkumar et al. 2011). Similarly, the LC_50_ and LC_90_ values against *Ae. aegypti* have been found to be 47.54 and 86.54ppm for *Mentha piperita*, 40.50 and 85.33ppm for *Zingiber officinale*, 115.60 and 193.30ppm for *Cu. longa* and, 148.50 and 325.70ppm for *Oc. basilicum*, respectively (Kalaivani et al. 2012) compared to 48.20 and 131.84ppm for *Co. grandis* in the present study. The LC_50_ and LC_90_ values of the *Co. grandis* leaf essential oils against larvae of *Cx. quinquefasciatus* were marginally better than essential oils of *Acorus calamus* reported by Senthilkumar and Venkatesalu (2012).

The results are also in agreement with several other previous reports where the major components of essential oils have shown excellent larvicidal or insecticidal activities, eg *Plectranthus amboinicus* leaf essential oil (Senthilkumar and Venkatesalu 2010), *Clausena anisata* leaf essential oil (Govindrajan 2011), *Feronia limonia* leaf essential oil (Senthilkumar et al. 2013). Similarly, Intirach et al. (2012) studied essential oils of six different plant families and demonstrated their larvicidal activity against laboratory colonized *An. cracens* mosquito. A careful observation of these representative studies indicated that there was no common thread in terms of chemical constituents, in these essential oils. The composition and major and minor components of essential oil are characteristics of particular plant and, at the best may be represented in the other members of same family. The composition and larvicidal activity of essential oil of a plant may vary as a function of age of plant, geographical location and season. The observed variations in the efficacy of essential oils from various plants against different vectors could be due to different chemical compositions and/or synergistic action of major and minor components in them (Senthilkumar and Venkatesalu 2012). The natural diversity of essential oils in the indigenous plants thus offer good opportunity of developing a cost effective, ecofriendly, region specific and practical strategy for the control of mosquito vectors either independently or as a part of integrated vector management strategy. Though there are no reports of insecticide/larvicide resistance in the study area, the same is well documented in African countries for *Anopheles* species against all the approved four classes (organochlorines, pyrethroids, carbamates and organophosphates) of insecticides (Kristan et al. 2003). Of the four classes, resistance to pyrethroids and its mechanisms in *An. gambiae*: the most important malarial vector in Africa has been extensively studied. It was found out that the insect develops resistance to insecticide either by altering its binding site, by point mutations, or by detoxifying it enzymatically before it reaches the target site (Tielong et al. 2014).

Pyrethroids are the insecticides of choice for mosquito control primarily because of their superior human and environment safety records (Adedayo et al. 2012). Besides, the use of insecticide mixtures and their periodic rotation, integrated vector management, involving biopesticides/essential oils of plant origins could be the answer for preventing/ delaying development of resistance in mosquitoes (Brogdon and Allister 1998).

The present study is a step forward in the direction, demonstrating the larvicidal potential of essential oil of a locally available plant against three most common mosquito species. The chemical analysis shows a different set of major and minor components in the essential oil than the earlier reported studies which can be gainfully utilized further.

## Conclusion

Results of this study will be helpful in developing cost effective, ecofriendly, region specific and practical strategy for the control of mosquito-borne diseases.

## References

[B1] Adedayo OO Emmanuel TI Muyiwa KO Adedapo OA Judith BO Olubunmi AO Taiwo SA (2012) Evidence of carbamate resistance in urban populations of *Anopheles gambiae* s.s. Parasit Vectors. 5: 116. 22686575 10.1186/1756-3305-5-116 PMC3409038

[B2] Ajay SS (2009) Hypoglycemic activity of *Coccinia indica* (Cucurbitaceae) leaves. Int J Pharm Tech Res. 1(3): 892–893.

[B3] Akpuaka A Ekwenchi MM Dashak DA Dildar A (2013) Biological Activities of Characterized Isolates of n-Hexane Extract of *Azadirachta indica* A. Juss (Neem) Leaves. New York Sci J. 6: 119–124.

[B4] Amer A Mehlhorn H (2006) “Larvicidal effects of various essential oils against *Aedes*, *Anopheles*, and *Culex* larvae (Diptera: Culicidae)”. Parasitol Res. 99: 466–472. 16642386 10.1007/s00436-006-0182-3

[B5] Anupam G Nandita C Goutam C (2012) Plant extracts as potential mosquito larvicides Indian J Med Res. 135(5): 581–598. 22771587 PMC3401688

[B6] Brogdon WG Allister JC (1998) Insecticide resistance and vector control. Emerg Infect Dis. 4(4): 605–613. 9866736 10.3201/eid0404.980410 PMC2640263

[B7] Cheng SS Chang HT Chang ST Tsai KH Chen WJ (2003) “Bioactivity of selected plant essential oils against the yellow fever mosquito *Aedes aegypti* larvae”. Bioresour Technol. 89: 99–102. 12676507 10.1016/s0960-8524(03)00008-7

[B8] Dharmagadda VSS Naik SN Mittal PK Vasudevan P (2005) Larvicidal activity of *Tagetes patula* essential oil against three mosquito species. Bioresour Technol. 96: 1235–1240. 15734310 10.1016/j.biortech.2004.10.020

[B9] Dua VK Pandey AC Raghavendra K Gupta A Sharma T Dash AP (2009) “Larvicidal activity of neem oil (*Azadirachtaindica*) formulation against mosquitoes”. Malar J. 8: 124. 19500429 10.1186/1475-2875-8-124 PMC2702347

[B10] Govindarajan M (2010) Chemical composition and larvicidal activity of leaf essential oil from *Clausena anisata* (Willd.) Hook. f. ex Benth (Rutaceae) against three mosquito species. Asian Pac J Trop Med. 3(11): 874–877.

[B11] Govindarajan M Sivakumar R Rajeswari M Yogalakshmi M (2010) Chemical composition and larvicidal activity of essential oil from *Mentha spicata* (Linn.) against three mosquito species. Parasitol Res. 110: 2023–2032. 10.1007/s00436-011-2731-7 22139403

[B12] Intirach J Choochote W Junkum A Chaithong U Champakaew D Tuetun B Jitpakdi A Pitasawat B (2012) Chemical constituents and combined larvicidal effects of selected essential oils against *Anopheles cracens* (Diptera: Culicidae) Psyche. 2012: 1–11.

[B13] Kalaivani K Senthil-Nathan S Murugesan AG (2012) Biological activity of selected *Lamiaceae* and *Zingiberaceae* plant essential oils against the dengue vector *Aedes aegypti* L. (Diptera: Culicidae). Parasitol Res. 110(3): 1261–1268. 21881945 10.1007/s00436-011-2623-x

[B14] Kannathasan K Senthilkumar A Venkatesalu V (2011) Mosquito larvicidal activity of methyl-p-hydroxybenzoate isolated from the leaves of *Vitex trifolia* Linn. Acta Trop. 120: 115–118. 21763671 10.1016/j.actatropica.2011.07.001

[B15] Kristan M Fleischman H Della TA Stich A Curtis CF (2003) Pyrethroid resistance/ susceptibility and differential urban/ rural distribution of *Anopheles arabiensis* and *A. gambiae* s.s malaria vectors in Nigeria and Ghana. Med Vet Entomol. 17: 326–332. 12941018 10.1046/j.1365-2915.2003.00449.x

[B16] Manas M Sunita Y Pawan K Raka K (2014) In vitro propagation and biosynthesis of steroidal sapogenins from various morphogenetic stages of *Moringa oleifera* Lam, and their antioxidant potential. Acta Physiol Plant. 36: 1749–1762.

[B17] Mittal PK Wijeyaratne P Pandey S (2004) Status of insecticide resistance of malaria, kala-azar and Japanese encephalitis vectors in Bangladesh, Bhutan, India and Nepal (BBIN), Environmental Health Project, Washigton, DC, USA.

[B18] Munasinghe MAAK Abeysena C Yaddehige IS Vidanapathirana T Piyumal KPB (2011) Blood Sugar Lowering Effect of *Coccinia grandis* (L.) J. Voigt: Path for a new drug for Diabetes Mellitus. Exp Diabetes Res. 2011: 978762. 21822423 10.1155/2011/978762 PMC3142553

[B19] Liu P Xin-Chao L Hui-Wen D Zhi-Long L Shu-Shan D Zhi-Wei D (2012) Chemical composition and insecticidal activity of the essential oil of *Illicium pachyphyllum* fruits against two grain storage Insects. Molecules. 17(12): 14870–14881. 23519259 10.3390/molecules171214870 PMC6268823

[B20] Rajkumar S Jebanesan A Nagarajan A (2011) Effect of leaf essential oil of *Coccinia indica* on egg hatchability and different larval instars of malarial mosquito *Anopheles stephensi*. Asian Pac J Trop Med. 4(12): 948–951. 22118029 10.1016/S1995-7645(11)60224-1

[B21] Ramaiah KD Das PK Michael E Guyatt H (2000) The economic burden of lymphatic filariasis in India. Parasitol Today. 16(6): 251–253. 10827432 10.1016/s0169-4758(00)01643-4

[B22] Rutledge CR Clarke F Curtis A Sackett S (2003) Larval mosquito control, Technical bulletin of the Florida mosquito control association. 4: 16–19.

[B23] Senthilkumar A Jayaraman M Venkatesalu V (2013) Chemical constituents and larvicidal potential of *Feronia limonia* leaf essential oil against *Anopheles stephensi*, *Aedes aegypti* and *Culex quinquefasciatus* Parasitol Res. 112: 1337–1342. 23160893 10.1007/s00436-012-3188-z

[B24] Senthilkumar A Venkatesalu V (2010) Chemical composition and larvicidal activity of the essential oil of *Plectranthus amboinicus* (Lour.) Spreng against *Anopheles stephensi*: a malarial vector mosquito. Parasitol Res. 107: 1275–1278. 20668876 10.1007/s00436-010-1996-6

[B25] Senthilkumar A Venkatesalu V (2012) Larvicidal potential of *Acorus calamus* L. essential oil against filarial vector mosquito *Culex quinquefasciatus* (Diptera: Culicidae). Asian Pac J Trop Med. 107: 1275–1278.

[B26] Senthilkumar A Verma P Gurusubamanian G (2009) Larvicidal and adulticidal activities of some medicinal plants against the malarial vector *Anopheles stephensi* (Liston). Parasitol Res. 104: 237–244. 18787842 10.1007/s00436-008-1180-4

[B27] Service MW (1996) Medical Entomology for Students. Chapman and Hall, London.

[B28] Siddiqui BS Rasheed M Ilyas F Gulzar T Tariq RM Naqvi SNH (2004) Analysis of Insecticidal *Azadirachta indica*. Z Naturforsch C. 59: 104–112. 15018062 10.1515/znc-2004-1-221

[B29] Singh G Kapoor IPS Singh P Heluani GSD Lampasona MPD (2008) Chemistry, antioxidant and antimicrobial investigations on essential oil and oleoresins of *Zingiber Officinale*. Food Chem Toxicol. 46: 3295–3302. 18706468 10.1016/j.fct.2008.07.017

[B30] Andreadis Theodore G Thomas Michael C John J (2005) Identification guide to the mosquito of Connecticut. Shepard Illustrations by Gale Ridge The Connecticut Agricultural Experiment Station.

[B31] Tielong X Daibin Z Linhua T Xuelian C Fengyang F Guiyun Y Bin Z (2014) *Anopheles sinensis* mosquito insecticide resistance: comparison of three mosquito sample collection and preparation methods and mosquito age in resistance measurements. Parasit Vectors. 7: 54. 24472598 10.1186/1756-3305-7-54 PMC3917893

[B32] World Health Organization (1981) Instruction for determining the susceptibility or resistance of mosquito larvae to insecticideas. WHO-VBC 81. 807: 1–6.

[B33] World Health Organization (2009) Global programme to eliminate lymphatic filariasis. Wkly Epidemiol Rec. 84: 437–444. 19860021

[B34] World Health Organization (2014) World Malaria Report. WHO, Geneva.

[B35] World Health Organization (2006) Guidelines for the Treatment of Malaria. WHO-WC. 770: 12–13.

[B36] Zhu BCR Henderson G Chen F Fei H Laine RA (2001) Evaluation of vetiver oil and seven insect-active essential oils against the Formosan subterranean termite. J Chem Ecol. 27: 1617–1625. 11521400 10.1023/a:1010410325174

